# High-Throughput UV Photoionization and Fragmentation of Neutral Biomolecules as a Structural Fingerprint

**DOI:** 10.3390/molecules28135058

**Published:** 2023-06-28

**Authors:** Siwen Wang, Yerbolat Dauletyarov, Daniel A. Horke

**Affiliations:** Institute for Molecules and Materials, Radboud University, 6525 AJ Nijmegen, The Netherlands

**Keywords:** mass spectrometry, photofragmentation, femtosecond ionization, multiphoton ionization, laser-based thermal desorption

## Abstract

We present UV photofragmentation studies of the structural isomers paracetamol, 3-Pyridinepropionic acid (3-PPIA) and (R)-(-)-2-Phenylglycine. In particular, we utilized a new laser-based thermal desorption source in combination with femtosecond multiphoton ionization at 343 nm and 257 nm. The continuous nature of our molecule source, combined with the 50 kHz repetition rate of the laser, allowed us to perform these experiments at high throughput. In particular, we present detailed laser intensity dependence studies at both wavelengths, producing 2D mass spectra with highly differential information about the underlying fragmentation processes. We show that UV photofragmentation produces highly isomer-specific mass spectra, and assign all major fragmentation pathways observed. The intensity-dependence measurements, furthermore, allowed us to evaluate the appearance intensities for each fragmentation channel, which helped to distinguish competing from consecutive fragmentation pathways.

## 1. Introduction

Mass spectrometry (MS) is one of the workhorses for molecular identification in analytical chemistry. Direct MS approaches, however, cannot distinguish isomers due to their identical molecular masses. Therefore, this requires more advanced approaches, such as additional spectroscopic information [1,2] or tandem-MS (MS/MS) with an intermediate fragmentation or reaction step [3]. In this scheme, the sample is first vaporized/ionized into the gas phase, followed by identification and mass selection of an ion of interest. This mass-selected ion is then fragmented using, for example, collision-induced fragmentation or photofragmentation, and the produced fragments are subsequently detected. We present here an alternative approach, in which the molecules of interest are introduced into the gas phase as intact neutral molecules. They are subsequently ionized and fragmented by a single femtosecond (UV) laser pulse and produced ions detected. By tuning the intensity of the laser pulse, this approach allows us to record detailed 2D maps of the observed fragmentation pattern as a function of laser intensity. We show that these maps are clearly molecular-structure-specific and allow one to distinguish isomers. By combining this single-MS technique with continuous molecular sources and high-repetition-rate lasers, this approach can, furthermore, provide a high throughput in a compact single-stage spectrometer.

Our approach relies on the introduction of intact biomolecules into the gas phase as neutral species. This is by itself an active area of research, which clearly still lags behind the production of gaseous charged biomolecules. The latter was revolutionized by approaches such as electrospray ionization (ESI) and matrix-assisted laser desorption-ionization (MALDI) [4]. The traditional approach for the vaporization of neutrals is simply heating of the sample in an oven, frequently followed by supersonic expansion with a carrier gas to produce a molecular beam. However, this approach is clearly not applicable to non-volatile and/or thermally labile systems, as with most biomolecules. This problem can be mitigated through the use of helium nanodroplets, which can be doped with (bio)molecules. Since the necessary vapor pressures in the employed pick-up cells are significantly lower then for a typical molecular beam source, thermal decomposition is significantly reduced [5].

A major advancement in the production of neutral gas phase biomolecules was the introduction of laser desorption jet cooling (LDJC), which rapidly desorbs molecules from a sample matrix, followed by immediate cooling within a supersonic expansion [6]. This approach has been very successful for desorbing a range of biomolecules, including nucleobases and nucleosides [7,8], as well as peptides and their aggregates [9,10]. The combination with supersonic expansion leads to rapid cooling, producing vibrationally and rotationally cold samples with manifold applications in high-resolution spectroscopy [11,12,13]. However, it has been shown that the laser desorption process can also lead to the production of a significant amount of fragments of the sample or matrix material, which are, hence, also entrained in the molecular beam [14,15]. This makes LDJC less suitable for studies utilizing so-called ‘universal probes’, which probe all molecules contained within a molecular beam. Examples of this include ultrashort pulse lasers [16,17] or X-ray/electron diffraction experiments [18,19]. These experiments require pure molecular beams, as do experiments specifically aimed at probing fragmentation processes, such as the one presented here. An additional disadvantage of all techniques relying on the production of a molecular beam through supersonic expansion is that this leads to a considerable gas load, and hence, requires extensive pumping capacity. This frequently limits the achievable densities or duty cycles of these experiments.

A recently developed alternative approach is laser-based thermal desorption (LBTD), which we utilize here [20]. This involves the application of sample to a thin (10 μm) Titanium substrate, which is subsequently heated in a controlled manner from the back side, leading to sample release into the gas phase. This leads to the production of intact neutral biomolecules as a high density and continuous molecular plume into the gas phase, enabling high repetition rate measurements on these samples with much-reduced vacuum pumping requirements [21]. The LBTD approach has now been successfully used for the desorption of nucleobases [21,22], nucleosides [20,23] and amino acids [17,24,25].

**Figure 1:**
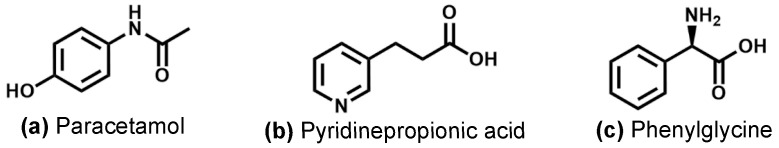
Molecular structures of paracetamol, 3-Pyridinepropionic acid and (R)-(-)-2-Phenylglycine.

In particular, we use this approach here to vaporize paracetamol, 3-Pyridinepropionic acid (3-PPIA) and (R)-(-)-2-Phenylglycine (Figure 1), which are all isomers with the structural formula $C_{8}H_{9}NO_{2}$, and hence, they have an identical molecular mass. Following desorption, molecules are ionized (and potentially fragmented) using femtosecond UV multiphoton ionization (fs-MPI) at 343 nm and 257 nm, and any produced ions were recorded. Thus, we present the first gas-phase-UV-photofragmentation studies of these target molecules. For all systems, we observed a clear signal from intact parent ions, again highlighting the softness of our approach. At elevated laser intensities, we observed significant photofragmentation of the target species, including several fragmentation channels that are isomer-specific, and hence, allow isomer identification. Furthermore, we recorded mass spectra as a function of incident laser intensity, yielding 2D mass spectra ‘heat maps’. These are found to be very sensitive to the underlying molecular structure, exhibiting large differences for the three isomers probed. This information can, furthermore, help to unravel the complex photofragmentation processes occurring in these systems.

## 2. Results

**Figure 2:**
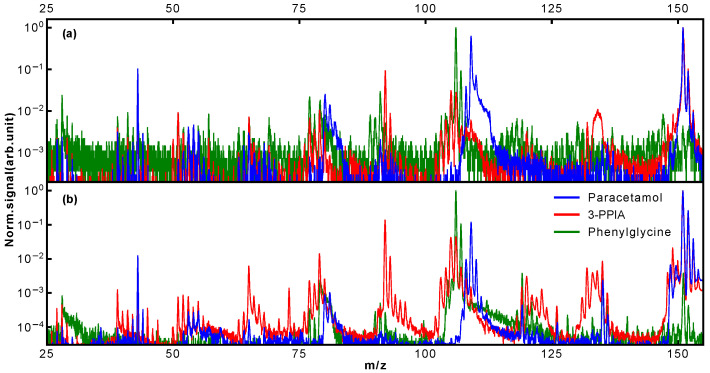
Normalized mass spectra of paracetamol, 3-PPIA and (R)-(-)-2-phenylglycine obtained using LBTD and UV femtosecond multiphoton ionization at (**a**) 343 nm and (**b**) 257 nm.

Typical mass spectra obtained for our three target systems following LBTD and fs-MPI at high laser intensity are shown in Figure 2 for ionization at 343 nm (2.2 $\times \;10^{11}$ W/cm${}^{2}$) and 257 nm (6.6 $\times \;10^{10}$ W/cm${}^{2}$); note the logarithmic intensity (*y*) axis. For better comparability, all spectra are normalized to the most intense peak, which corresponded to the parent ion for 3-PPIA and paracetamol, and the m/z 106 fragment for phenylglycine, as further discussed below.

The ionization energy of paracetamol is well known and was experimentally determined to be 7.57 eV [26]. Therefore, ionization requires a minimum of three photons of 343 nm (total energy 10.83 eV) or two photons at 257 nm (total energy 9.64 eV). The corresponding ionization potentials for 3-PPIA and phenylglycine are less well known. In the case of 3-PPIA, only a theoretical value of 7.24 eV is available in the literature [27], which is very close to the total photon energy of two 343 nm photons (7.22 eV). However, as will be discussed further below, our results indicate that 3-PPIA also requires three photons of 343 nm, or two at 257 nm. For phenylglycine, no published values for the ionization energy are available; a reasonable estimate might be the appearance potential of parent ions, which was determined to be around 8.9 eV [28]. This again indicates a requirement of three photons of 343 nm or two photons of 257 nm for ionization.

**Figure 3:**
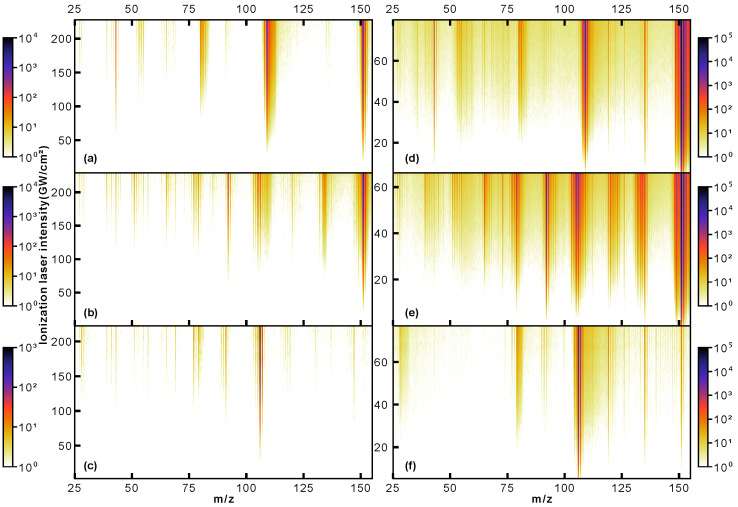
Mass spectra of paracetamol (**a**,**d**), 3-PPIA (**b**,**e**) and phenylglycine (**c**,**f**) as a function of incident ionization laser intensity, producing 2D mass spectra. Note the logarithmic intensity (*z*) color scale. The left column (**a**–**c**) corresponds to 343 nm ionization, and the right column (**d**–**f**) to 257 nm ionization.

The mass spectra presented in Figure 2 already show clear differences between isomers. Most notably, for phenylglycine, the observed relative parent ion signal is significantly less, around three orders of magnitude, than for the other species. In contrast to this, for both paracetamol and 3-PPIA, the intact parent ion at 151 Da is the most intense peak observed. Of the molecular fragmentation channels, it is in particular the peaks around 103–110 Da, 90–94 Da and 73–81 Da that show structure-specific signatures, as will be discussed further below. In order to increase the information content we collected mass spectra at a range of ionization laser intensities, producing two-dimensional ‘heat maps’ of the observed mass spectra as a function of incident laser intensity [21]. These are shown in Figure 3 for the three target species at 343 nm (left column) and 257 nm (right column), with the *y*-axis representing the ionization laser intensity, and the observed ion counts are shown on a logarithmic color (*z*) scale. These data contain detailed information about different fragmentation pathways and at which incident intensities these become available, and shows very visually the different, and molecular-structure-specific, fragmentation behavior.

## 3. Discussion

**Figure 4:**
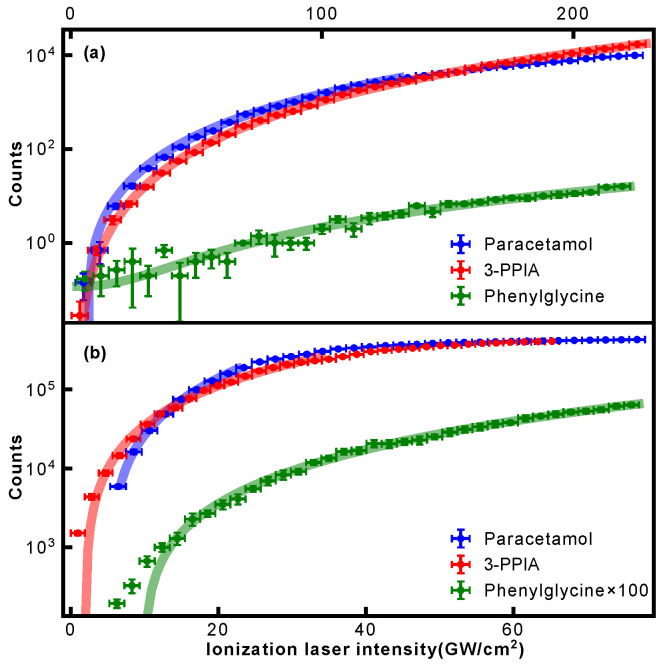
Observed parent ion signal (data points) for paracetamol, 3-PPIA and phenylglycine as a function of ionization laser intensity for (**a**) 343 nm and (**b**) 257 nm. Solid lines correspond to a power law fit to the data.

The intensity dependence shown in Figure 3 allowed us to evaluate the photon order of the occurring ionization processes. For this, the intensity dependence of the respective parent ion signal was evaluated and fitted with a power law of the form $y=ax^{n}+b$. Resulting fits are shown in Figure 4, and the extracted exponents summarized in Table 1. This confirms that at 343 nm, at least three photons are required for ionization for all samples. For 257 nm ionization, both paracetamol and phenylglycine show exponents close to 2, thus indicating a non-resonant 2-photon ionization. A previous study on paracetamol by Beames et al. also observed no resonances in this wavelength range [29]. For 3-PPIA, however, a much smaller exponent of 1.6 is observed, indicating the involvement of a resonant step, and hence, likely a resonance-enhanced two-photon ionization. Recent theoretical calculations of the gas-phase UV absorption spectrum of 3-PPIA found a very broad (∼30 nm) absorption in the UV and centered at 261 nm [27], consistent with our observations. This was attributed mostly to the HOMO→LUMO transition. Resonance enhancement is, moreover, consistent with the overall much larger fragmentation yield of 3-PPIA at 257 nm, compared to both phenylglycine and paracetamol. Most likely, the resonance enhancement also increases the probability of absorbing additional 257 nm photons after ionization, leading to enhanced fragmentation through so-called ‘ladder climbing’ [30].

**Figure 5:**
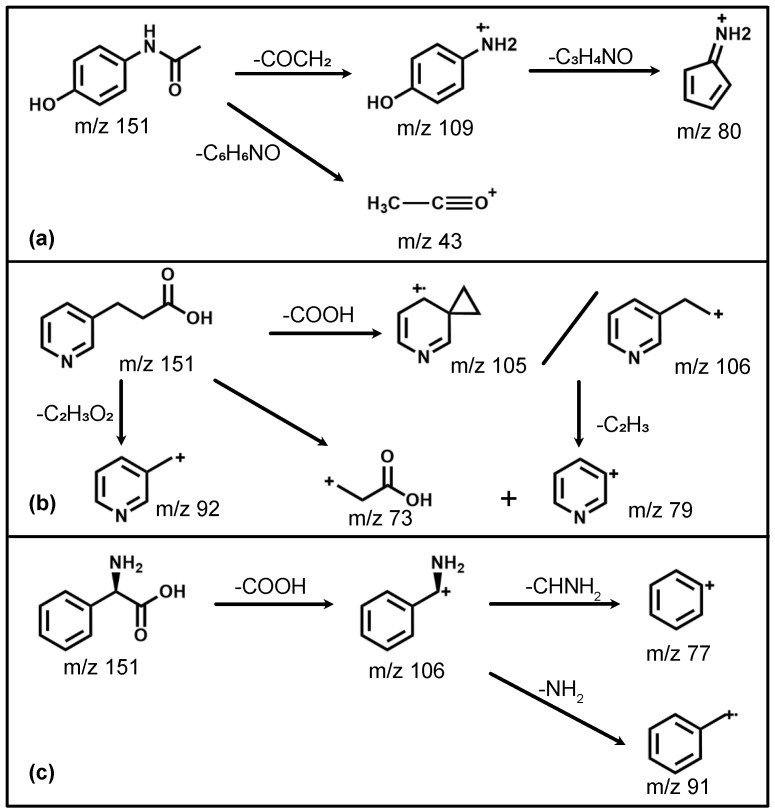
Overview of the major photofragmentation pathways observed for (**a**) paracetamol [31,32], (**b**) 3-PPIA [33] and (**c**) phenylglycine [28,33,34]. See text for further details.

We will now discuss the isomer-specific fragmentation observed for three distinct m/z regions (103–110 Da, 90–94 Da and 73–81 Da); the assigned fragmentation channels are also summarized in Figure 5. The observed fragmentation patterns are overall qualitatively similar for both employed wavelengths. This is consistent with non-resonant ionization processes, which should depend only on the total available energy. The discussion that follows, hence, also pertains to both wavelengths used.

**Figure 6:**
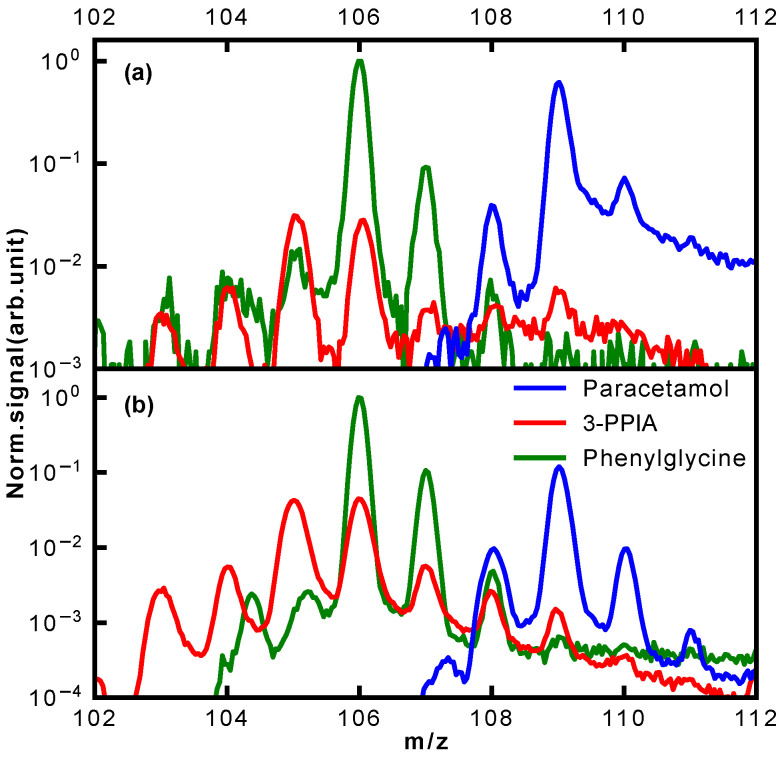
Normalized mass spectra of the three isomers in the mass range of m/z 102–112 Da following ionization at (**a**) 343 nm and (**b**) 257 nm for the highest ionization laser intensities used.

For the mass range 103–110 Da, a remarkable difference in the photofragmentation for the three isomers is observed; a close-up of this is shown in Figure 6. This mass range represents the major fragmentation channel observed for paracetamol and phenylglycine. For paracetamol, we predominantly see formation of a 4-aminophenol cation (109 Da) through loss of an acetyl group, as suggested by a previous photolysis study [31]. The leaving acetyl group is also clearly observed at 43 Da. Moreover, both these ions have an onset at similar ionization laser intensities, in particular for ionization at 257 nm (Figure 3d). This suggests that they, indeed, stem from the same fragmentation channel. This channel is not available for both 3-PPIA and phenylglycine. For the latter, the fragment at 106 Da dominates, corresponding to loss of the carboxylic acid group, consistent with previous experiments [28,34]. The same channel is also observed for 3-PPIA. Moreover, we also observe significant intensity for the 105 Da fragment, which we assign to the formation of a cyclopropyl group. This is consistent with assignments from collision-induced dissociation studies of deprotonated 2-Pyridinepropionic acid [33].

**Figure 7:**
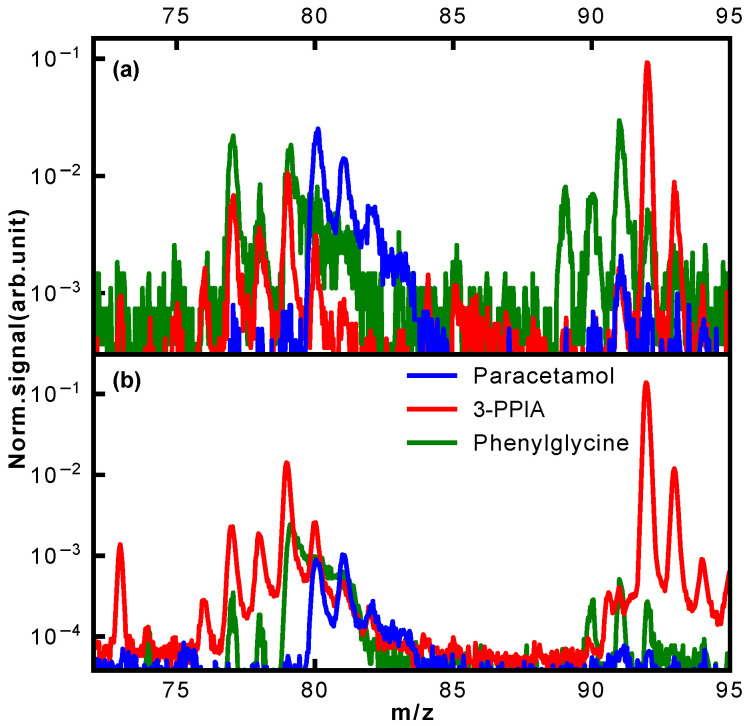
Normalized mass spectra of the three isomers in the mass range of m/z 72–95 Da following ionization at (**a**) 343 nm and (**b**) 257 nm for the highest ionization laser intensities used.

A further clear difference in fragmentation is observed in the mass region of 90–94 Da, shown in more detail in Figure 7. Whereas hardly any fragments are observed for paracetamol in this mass region, both phenylglycine and 3-PPIA show significant counts in this range, peaking at 91 Da for phenylglycine and 92 Da for 3-PPIA. We assign this to formation of a benzyl (phenylglycine) or methylpyridine (3-PPIA) cation following cleavage of the side chain, as illustrated in Figure 5. The presence of the pyridine ring in 3-PPIA here leads to a clear mass separation of the observed fragments from the two isomers. For both 3-PPIA and phenylglycine we furthermore observe the formation of benzene and benzyl fragments at m/z of 78 Da and 90 Da, respectively, corresponding to side chain loss. As expected, these are absent for paracetamol. Interestingly, we see no evidence for the formation of phenol fragments (94 Da) from paracetamol.

Similarly, considerable differences between the isomers were observed in the mass region 73–81 Da, also shown in Figure 7. For 3-PPIA, we see a clear signal at 73 Da, which we assign to side chain loss, and hence, the formation of a (deprotonated) propionic acid cation. This channel is clearly not available to both paracetamol and phenylglycine. In contrast to this, phenylglycine predominantly yields fragments of 77 Da, again corresponding to side chain loss and formation of a phenyl cation. For paracetamol, on the other hand, the predominant fragment produced in this mass range is at 80 Da. Following the assignment made in a previous electron impact ionization study [32], we believe this to be due to the formation of a cyclopentadienylidene ammonium cation through loss of formaldehyde from the 109 Da fragment, as shown in Figure 5.

The observed fragmentation patterns, hence, show distinct isomer-specific features. They can, therefore, be considered a structural fingerprint that allows species identification, for example, via comparison to reference spectra. Distinct fragment peaks can, moreover, be related to particular functional groups present; for example, the presence of a pyridine ring leads to distinct fragments at m/z 92 (methylpyridine), as observed for 3-PPIA only. Similarly, the amide group present in paracetamol leads to acetyl fragments (m/z 43) that are absent for the other species. Thus, even if a reference spectrum is not available, signifcant structural insight can still be gained from these fragmentation mass spectra.

Further fragmentation channels, but which do not show a particular isomer-specific sensitivity, were observed in the mass range 133–135 Da. We assign this to the effective loss of O, OH or $H_{2}O$; these fragments are also observed in their respective reference electron impact ionization spectra [35]. Compared to both paracetamol and phenylglycine, 3-PPIA showed significantly enhanced fragmentation at 257 nm, which we attribute to the resonance-enhanced process, as discussed above. In particular, additional fragmentation channels around 51 Da and 65 Da were observed, which we assign to further fragmentation of the aromatic ring systems, leading to formation of, among others, prominent peaks at 51 Da ($C_{4}H_{3}^{+}$) and 65 Da ($C_{5}H_{5}^{+}$).

**Figure 8:**
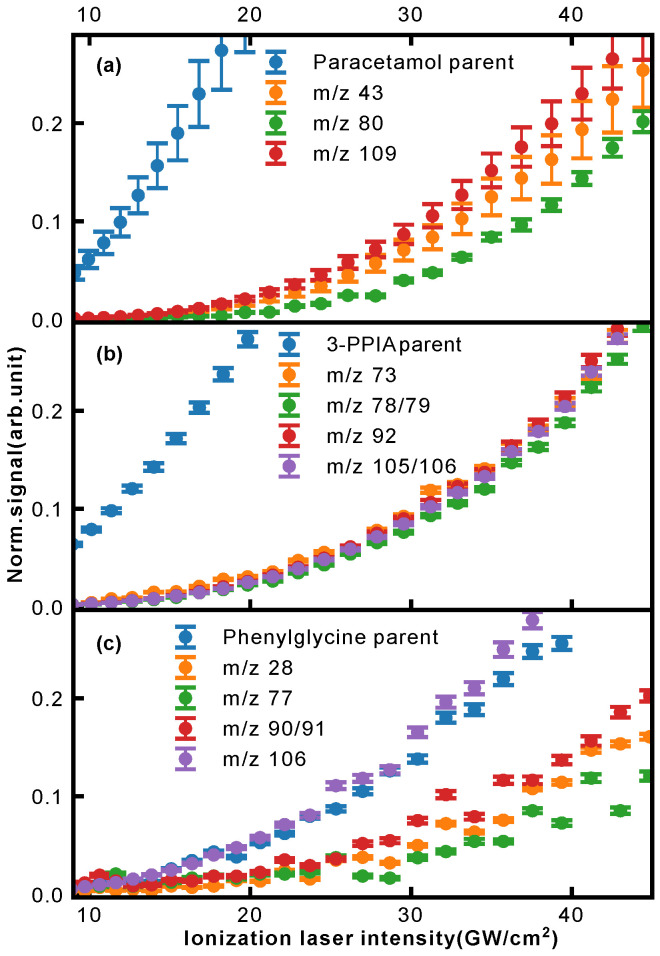
Normalized ion intensities for the parent and selected fragment ions of (**a**) paracetamol, (**b**) 3-PPIA and (**c**) phenylglycine as a function of incident laser intensity for 257 nm.

Further insight into the occurring fragmentation pathways can be gained by taking into account the ionization laser intensity dependence of the different fragment masses, contained in the two-dimensional mass spectra of Figure 3. In particular, the appearance energy carries information about when fragmentation channels open, and can give an indication of whether particular channels compete or are occurring consecutively. In Figure 8, we show the normalized ion signal as a function of ionization laser intensity (here for 257 nm light) for several fragments of paracetamol (a), 3-PPIA (b) and phenylglycine (c).

In the case of 3-PPIA (Figure 8b), we saw that all fragments have a significantly higher appearance intensity than the corresponding parent ion. Moreover, all fragments showed very similar behavior indicating that these are not consecutive, but rather competing processes. Given the fact that for 3-PPIA we excite a resonance in the molecule [27], it appears that two-photon absorption predominantly produces the parent ion, and all further fragmentation channels are enabled by the absorption of an additional UV photon. This is confirmed by the photon order (power law exponent) for the fragments, which were found to be 3.1 (106 Da) and 3.0 (92 Da), compared to 2.0 for the parent ion.

Both paracetamol and phenylglycine showed very different behavior. For paracetamol (Figure 8a), we again saw a much earlier appearance intensity for the parent ion. However, in contrast to 3-PPIA, the fragments showed differing behavior with increasing laser intensity. The primary photofragments of mass 109 Da and 43 Da, corresponding to loss of the acetyl group (Figure 5), appeared first, and further fragmentation to form the 80 Da product only appeared at higher intensities, indicating consecutive fragmentation processes. For phenylglycine, both the parent ion and primary photoproduct at 106 Da were observed at very similar appearance intensities, indicating that the parent cation is likely unstable with a high propensity for direct fragmentation via loss of the carboxylic acid group. This also explains the overall much lower parent ion yield observed for phenylglycine, which was also the case for reference electron impact ionization mass spectra [35]. It is only at much higher intensities that further fragmentation channels open up, first the production of benzyl (91 Da) and at then even higher intensities of phenyl (77 Da) fragments, again pointing to consecutive fragmentation processes.

## 4. Materials and Methods

**Figure 9:**
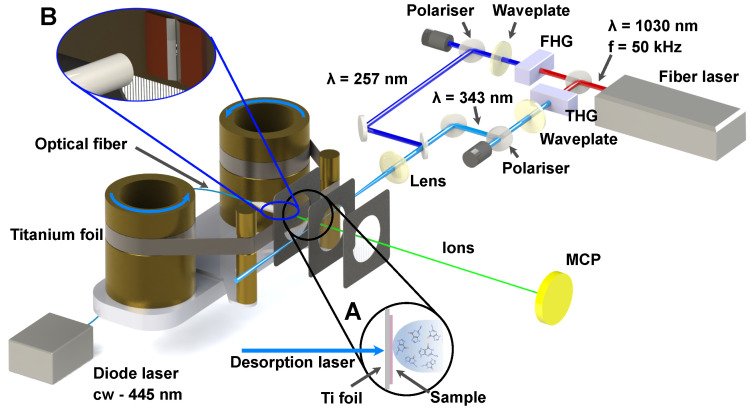
Overview of the experimental setup. Sample is deposited on a 10 μm titanium foil band (inset A), which is constantly moved at 25 μm/s to provide fresh sample. Desorption is achieved by irradiating the backside of the foil with a continuous fiber-coupled 445 nm laser diode (inset B). Once desorbed, sample is ionized inside a Wiley–McLaren time-of-flight setup by femtosecond laser pulses generated by third-harmonic generation (THG, 343 nm) and fourth-harmonic generation (FHG, 257 nm) from the fundamental centered at 1030 nm. Produced ions are accelerated towards a microchannel plate detector (MCP), where single ion hits are detected, time-stamped and fed to a computer for further analysis.

Our LBTD-coupled mass spectrometer has been described previously [21,36], and we focus here only on details pertinent to the current study. All samples were purchased from Sigma Aldrich and used without further purification. Target molecules were put into an aqueous solution (0.013 M) and applied onto the surface of a 10 μm thick titanium foil using a commercial airbrush gun. After drying, the foil was mounted into the LBTD source (Figure 9), consisting of two rollers. During data collection, these were rotated at a velocity of 25 μm/s to continuously provide fresh sample in the interaction region. The sample was desorbed by irradiating the back side of the titanium foil with a continuous diode laser (445 nm) (see inset A in Figure 9), and the irradiated area on the foil was limited to 3 mm × 0.2 mm (inset B of Figure 9). Employed desorption laser powers were 27 mW (paracetamol), 102 mW (phenylglycine) and 63 mW (3-PPIA).

Following desorption, samples were ionized by the third (343 nm) or fourth (257 nm) harmonic of an ytterbium-doped fiber femtosecond laser (Active Fiber Systems GmbH). Typical pulse durations were of the order of 250 fs (FWHM), and the laser was operated at 50 kHz repetition rate. The beam was focused into the interaction region using a plano-convex lens with a nominal focal length f=500 mm to a spot size of around 200 μm, as measured by a knidge-edge scan. The intensity in the interaction region was controlled using variable attenuators, consisting of a motor-controlled half-wave plate and a thin-film polarizer.

Produced ions were detected in a custom-built Wiley–McLaren time-of-flight mass spectrometer with a typical mass resolution $\frac{m}{\Delta m}∼500$, operated in ion counting mode using a constant fraction discriminator (Surface Concept GmbH) and time-to-digital converter (cronologic GmbH). This leads to a typical dynamic range $>10^{5}$. Each spectrum was collected for $3\times 10^{5}$ laser shots, corresponding to ∼6 s of collection time at 50 kHz.

## 5. Conclusions

By combining laser-based thermal desorption and fs-MPI, we have reported the first UV-photofragmentation studies of paracetamol, 3-PPIA and phenylglycine at wavelengths of 343 nm and 257 nm. In all cases, three photons of 343 nm and two photons of 257 nm were required for ionization, which was a non-resonant process with the exception of 3-PPIA at 257 nm, where we observed a clear resonance enhancement. Despite these molecules all being isomers with the structural formula $C_{8}H_{9}NO_{2}$, clear structure-specific fragmentation behavior for each molecule was observed. We have, furthermore, recorded data for a wide range of different laser intensities, producing 2D mass spectra that clearly show intensity onsets for different fragmentation channels. This allowed us to differentiate competing (3-PPIA) from consecutive (paracetamol and phenylglycine) fragmentation pathways. We have, hence, demonstrated the collection of multidimensional structure-specific data, which can serve as a ‘structural fingerprint’ for mass-spectrometry based isomer identification.

The combined approach of LBTD with fs-MPI is clearly a very powerful approach of introducing intact biomolecules into the gas phase at high densities. It also has the potential for vaporizing significantly larger and more fragile systems intact, as has recently been demonstrated [37]. The continuous nature of the source allows the combination with high repetition-rate lasers for high throughput experiments with the overall repetition rate, and hence, throughput limited by the ion time-of-flight. At the 50 kHz repetition rate utilized in this study, for example, the entire intensity scan shown in Figure 3a, consisting of 61 distinct laser intensities, can be collected in less than 6 min. While the produced samples are not cooled to cryogenic internal temperatures, the purity of the produced ‘molecular plume’ nonetheless opens up plenty of applications for experiments that require pure samples, such as femto/attosecond dynamics experiments or diffractive molecular imaging approaches.

## Figures and Tables

**Table 1:** Exponents (photon orders) extracted from the power law fitting shown in Figure 4 for both 343 nm and 257 nm.

Wavelength	343 nm	257 nm
Paracetamol	2.9 ± 0.1	2.0 ± 0.1
3-PPIA	3.4 ± 0.1	1.6 ± 0.1
Phenylglycine	2.6 ± 0.3	2.0 ± 0.1

## Data Availability

Data are available from the corresponding author upon reasonable request.

## Sample Availability

Samples of the compounds are commercially available.
